# PHDs-seq: a large-scale phenotypic screening method for drug discovery through parallel multi-readout quantification

**DOI:** 10.1186/s13619-023-00164-9

**Published:** 2023-06-02

**Authors:** Jun Li, Jun Chi, Yang Yang, Zhongya Song, Yong Yang, Xin Zhou, Yang Liu, Yang Zhao

**Affiliations:** 1 State Key Laboratory of Natural and Biomimetic Drugs, MOE Key Laboratory of Cell Proliferation and Differentiation, Beijing Key Laboratory of Cardiometabolic Molecular Medicine, Institute of Molecular Medicine, College of Future Technology, Peking University, Beijing, 100871 China; 2 Peking-Tsinghua Center for Life Sciences, Peking University, Beijing, 100871 China; 3 Plastech Pharmaceutical Technology Ltd, Nanjing, 210031 China; 4 Jiangsu Key Laboratory of Molecular Biology for Skin Diseases and STIs, Institute of Dermatology, Chinese Academy of Medical Sciences and Peking Union Medical College, Nanjing, China; 5 Department of General Surgery, Peking University Third Hospital, Beijing, 100191 China

**Keywords:** High-throughput screening, PHDs-seq, Adipocyte reprogramming, ABT869

## Abstract

High-throughput phenotypic screening is a cornerstone of drug development and the main technical approach for stem cell research. However, simultaneous detection of activated core factors responsible for cell fate determination and accurate assessment of directional cell transition are difficult using conventional screening methods that focus on changes in only a few biomarkers. The PHDs-seq (Probe Hybridization based Drug screening by sequencing) platform was developed to evaluate compound function based on their transcriptional effects in a wide range of signature biomarkers. In this proof-of-concept demonstration, several sets of markers related to cell fate determination were profiled in adipocyte reprogramming from dermal fibroblasts. After validating the accuracy, sensitivity and reproducibility of PHDs-seq data in molecular and cellular assays, a panel of 128 signalling-related compounds was screened for the ability to induce reprogramming of keloid fibroblasts (KF) into adipocytes. Notably, the potent ATP-competitive VEGFR/PDGFR inhibitor compound, ABT869, was found to promote the transition from fibroblasts to adipocytes. This study highlights the power and accuracy of the PHDs-seq platform for high-throughput drug screening in stem cell research, and supports its use in basic explorations of the molecular mechanisms underlying disease development.

**Supplementary Information:**

The online version contains supplementary material available at 10.1186/s13619-023-00164-9.

## Background

Phenotypic screening methods has greatly accelerated the study of mechanisms involved in cell fate transition, and additionally has enabled the development of new chemical tools for stem cell research (Vandana et al. 2021). However, the traditional assays mainly use a reporter system or immunostaining to focus on very few biomarkers or a single phenotype, such as cell proliferation, viability, morphology (Vandana et al. 2021), which are too inaccurate to assess cell fates or states, and thus result in high false positive rates. In comparison, transcriptional profiling could be an improved strategy for phenotyping in stem cell and cell reprogramming research (Buganim et al. 2012; Hu et al. 2022; Joung et al. 2023), since mRNA profiling-based approaches have several essential advantages over conventional strategies in that they measure cell identity/state, through broad assessment of the expression of all relevant genes, rather than the effects in very limited markers. More specifically, the advantages of mRNA profiling can be divided into three distinct categories: 1) transcriptional profiling more accurately reflects the integrity and quality of an induced cell fate and more comprehensively infers unintentional transition tendencies; 2) transcriptional profiling can simultaneously detect the activation of each individual core transcription factor known to determine cell fate, thereby providing small molecule candidates with different mechanisms of action (MOA) for further combination; 3) transcriptional profiling can also detect side effects of candidate drugs. In addition, transcriptional profiling screens can lead to original biological discoveries, such as new drug targets and mechanisms (Ye et al. 2018). However, transcriptional profiling strategies, such as RNA-sequencing and qPCR, are cost-ineffective and labor-intensive, impairing their application for high-throughput chemical screen.

In recent years, high-throughput sequencing platforms have enabled several revolutionary advances in phenotypic and functional screening, such as DRUG-seq and PLATE-seq, which use genome-wide transcriptional profiling with hierarchical clustering and MOA analysis to screen out effective compounds (Bush et al. 2017; Ye et al. 2018). L1000-based connectivity mapping infers mRNA profiles through transcript representation in a set of 978 marker genes in a panel cancer cell lines (Subramanian et al. 2017). L1000 can thus provide an abundance of transcriptional information about the effects of multiple chemical compounds and targets in various cell lines.

In addition, several targeted sequencing methods based on probe hybridization and ligation-dependent amplification have been reported over the past two decades (Akers et al. 2011; Kondrashova et al. 2015; Larman et al. 2014; Schouten et al. 2002; Teder et al. 2018; Wang et al. 2017; Yeakley et al. 2017; Zhang et al. 2015). These approaches can reveal previously unrecognized mechanisms of candidate drugs in different cancers and diseases (Theodoris et al. 2020; Wang et al. 2021). Targeted sequencing has the advantages of only detecting relevant signatures while excluding irrelevant profiles and reducing costs dramatically. In general, although previously reported targeted sequencing approaches have been considered more suitable for largescale screens, they are accompanied by some limitations, such as labor intensive protocols, insufficient cost-effectiveness and time saving, inaccurate or biased quantification due to uneven PCR amplification, or heavy reliance on customized reagents (Li et al. 2012).

Keloids are abnormally large scars in skin tissue caused by aberrant collagen deposition from activated fibroblasts. Despite the availability of many therapeutic interventions, including surgical (e.g., through laser resection) and pharmacological strategies, that are widely applied in clinic, preventing the recurrence of fibrosis persists as a long-term challenge. Myofibroblasts spontaneously transform into adipocytes during wound healing in mice, which can be beneficial for fibrosis through upregulation of *PRDM16* and *UCP1* (Cohen and Kajimura 2021; Henderson et al. 2020). Moreover, adipocytes are essential for fibroblast recruitment and dermal reconstruction during wound healing (Schmidt and Horsley 2013). Specifically, fatty acid oxidization and PPARγ signalling can promote the degradation of extracellular matrix (ECM) (El Agha et al. 2017; Zhao et al. 2019). Thus, reprogramming myofibroblasts (keloid fibroblasts) to form adipocytes represents a promising strategy to prevent myofibroblast proliferation and collagen deposition. Previous studies have reported that keloid-associated dermal fibroblasts can be reprogrammed into adipocytes in vitro using specialized adipocyte differentiation (AD) medium containing a set of small molecules (1% ITS, 0.5 mM isobutylmethylxanthine, 0.1 μM cortisol, 1 μM dexamethasone, 0.2 nM triiodothyronine, 1 μM rosiglitazone) and the cytokine BMP4 (Plikus et al. 2017). Moreover, inhibitors of the TGFβ receptor, such as 616,452, can improve brown adipogenesis and browning of white adipocytes in the presence of rosiglitatone (Tu et al. 2019). However, systematic evaluation of the efficacy of these small molecules in reprogramming keloid-associated myofibroblasts into adipocytes is still necessary before clinical testing of this alternative in situ reprogramming strategy for keloids.

In this study, we developed a highly integrated drug screening platform, PHDs-seq (Probe Hybridization based Drug screening by sequencing) to overcome some limitations inherent in previous high-throughput mRNA profiling methods. PHDs-seq shows relatively high sensitivity, accuracy, and reproducibility, indicating its suitability for screening chemical compounds that induce cell reprogramming. Through drug screening with PHDs-seq, we found that ABT869, a novel and potent ATP-competitive VEGFR/PDGFR inhibitor of the KDR (Kinase insert Domain Receptor), promotes adipocyte reprogramming from skin dermal myofibroblasts.

## Results

### Establishment of PHDs-seq platform

**Fig. 1:**
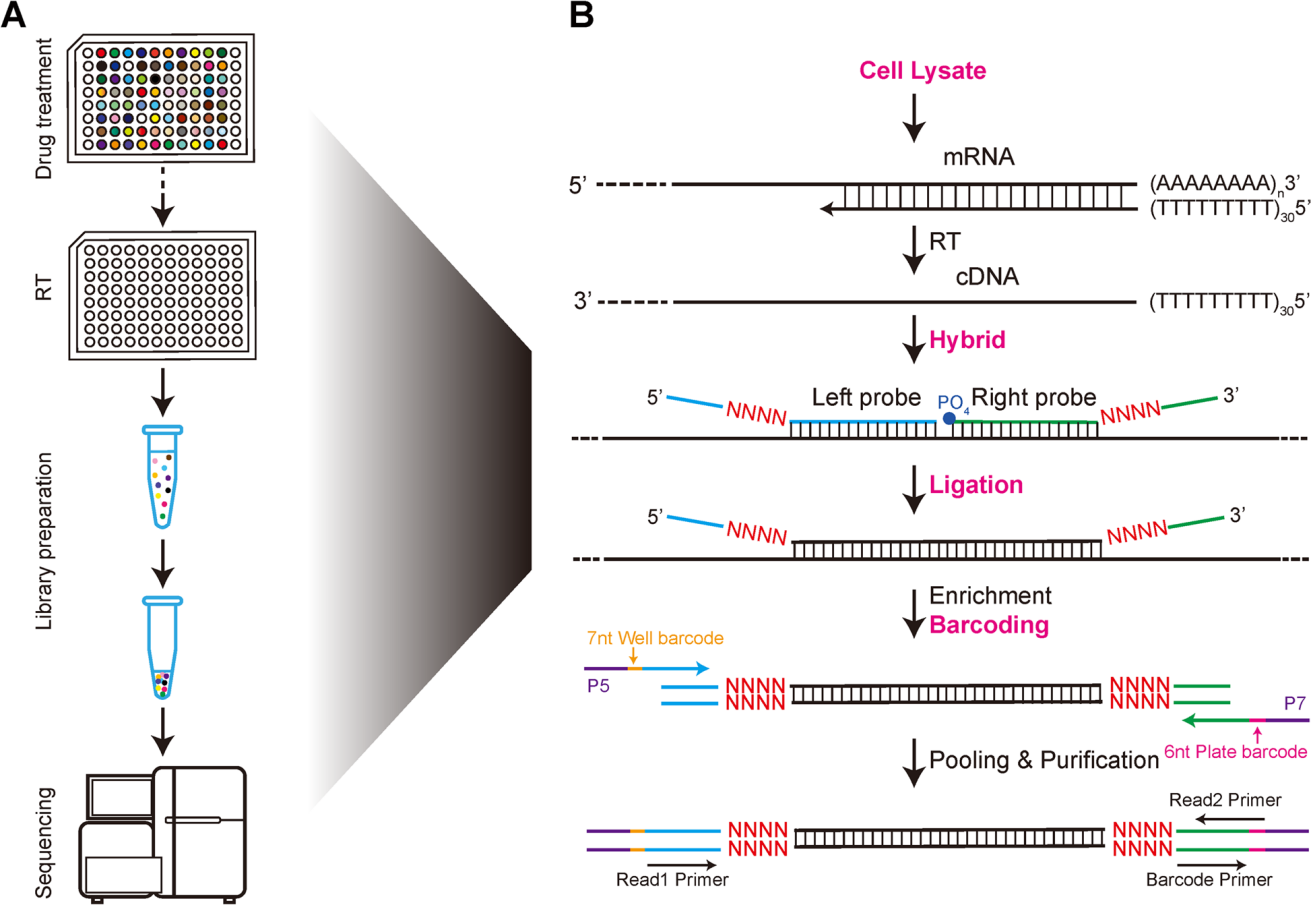
PHDs-seq library preparation flow chart **A** and library procedures **B**

To measure the expression of specific genes of interest, we developed the PHDs-seq platform (Fig. 1). Briefly, cells growing in 96-well plates were lysed and mRNAs were reverse transcribed into cDNA using oligo-dT_30_ primer. A mixture of probe pairs, each with a 4nt unique molecular identifier (UMI) in the left and right probes, was then added to hybridize with the newly synthesized cDNA. Simultaneously, the probe pairs hybridizing to the same cDNA template ligated with each other through a phosphodiester bond between the 5’-phosphate in right probe and the 3’-hydroxyl in left probe, producing a UMI-left probe-right probe-UMI product. The ligation products were then captured using magnetic beads and amplified by PCR using 7nt P5 well and 6nt P7 plate barcoded primers to extend throughput. The PCR products were pooled together and again purified with magnetic beads. Finally, the purified libraries were subjected to quality control and concentration detection prior to sequencing on the Illumina Hiseq X-ten platform.

### Systematic evaluation of PHDs-seq

To test whether PHDs-seq could detect adipocyte induction in the early stages of reprogramming primary skin dermal myofibroblasts, at day 5 (D5) and day 8 (D8) of induction, we collected cells lacking identifiable adipocyte lipid characteristics in order to detect transcriptomic signatures of fibrosis or adipocytes. For this purpose, gene probes related to each respective signature were prepared targeting cell identity-specific markers (Fig. S1A and Table S1). Fibroblast markers included *PRRX1* and *THY1,* common fibroblast markers; *ACTA2,* a marker of fibroblast activation; *FBN1, COL1A1,* and *COL3A1,* components of extracellular matrix that are highly expressed in fibrotic cells; *MMP1,* a marker involved in the breakdown of extracellular matrix; and *TIMP1,* an inhibitor of *MMP1* activity. Adipocyte signature markers included *FABP4, ADIPOQ, EBF2, CEBPA,* and *ATF2,* as well as *ZNF423* and *LEP,* markers of white adipocytes; *ZNF516, PRDM16, PPARG, PPARGC1A, FNDC5,* and *UCP1,* markers of brown adipocytes; and functional markers such as the insulin receptor *INSR,* which relays the insulin signals to regulate lipid synthesis and storage, and *SLC2A4*, an insulin-responsive glucose transporter. Further, to ensure that sufficient number of reads were available for each marker, especially low abundance transcripts, probes for eight housekeeping genes were added (*ACTB, CYC, GAPDH, HMBS, PPIA, SDHA, TBP,* and *YWHAZ*) which were previously shown to range widely in expression level (Teder et al. 2018). To test the accuracy of PHDs-seq, two pairs of probes were designed for *PRDM16, UCP1, EBF2, ADIPOQ, COL1A1, ACTB, GAPDH, and THY1*. The 49 total probe pairs were then pooled and applied to quantify mRNA levels of each gene in the induced fibroblasts.

**Fig. 2:**
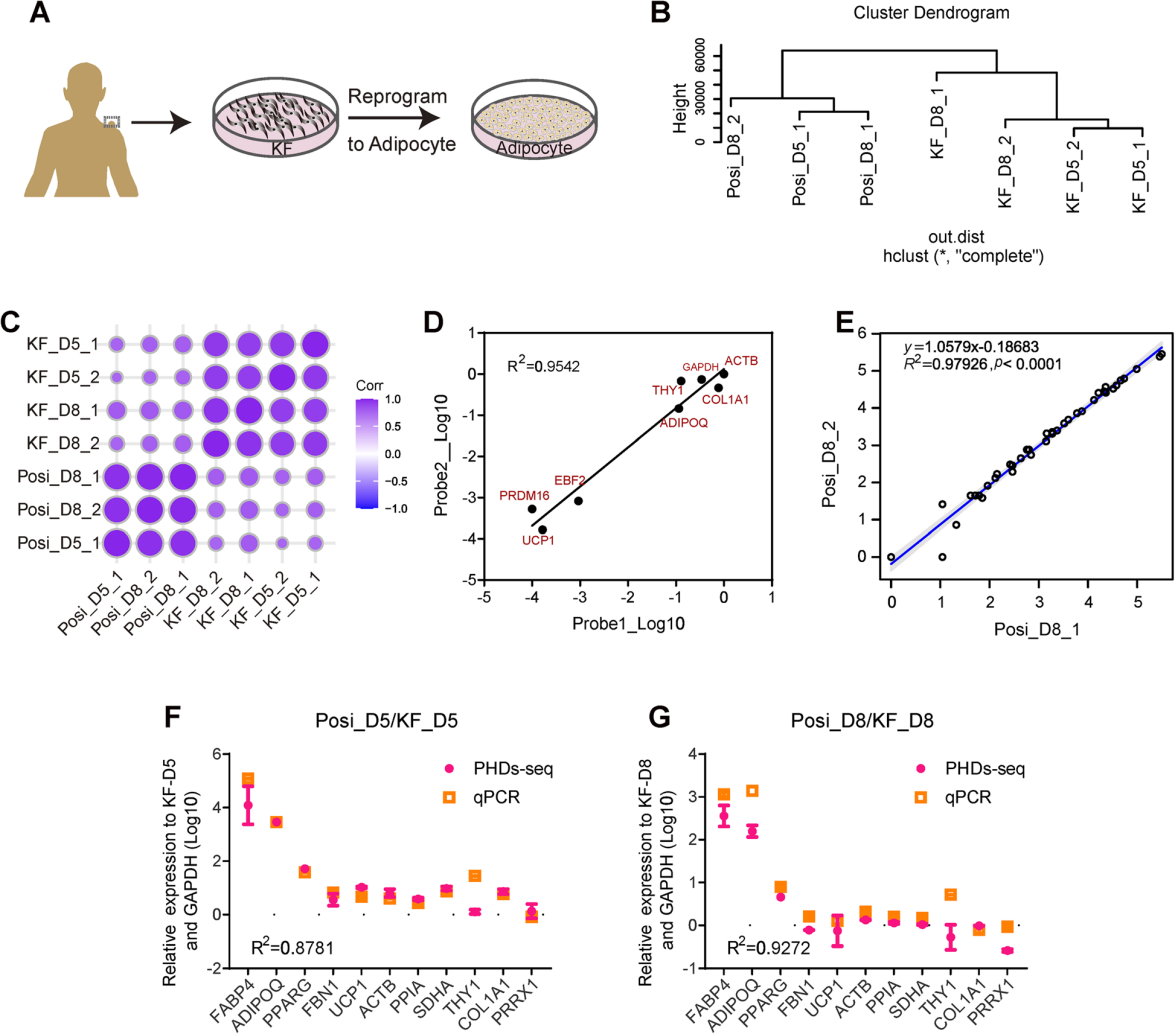
Systematic evaluation of PHDs-seq. **A** Schematic diagram for KF-adipocyte reprogramming. **B** Clustering analysis of PHDs-seq data from positive and negative samples. Posi: keloid fibroblasts cultured in AD medium; KF: keloid fibroblasts cultured in KF medium; D5: day5; D8: day8; _1 and _2 represent two biological replicates. **C** Pearson correlation analysis of PHDs-seq data from positive and negative samples. **D** Validation of probe performance using redundant probe pairs for a single gene in PHDs-seq. **E** Validation of consistency between biological replicates in PHDs-seq. **F**-**G** Comparison between PHDs-seq performance and qPCR assays in quantifying relative mRNA levels in samples collected at D5 (F) and D8 (G)

After library preparation, quality control by agarose gel electrophoresis and capillary electrophoresis confirmed that library fragment size was ~ 210 bp, which was consistent with the expected size of 208 bp (Fig. S1B and C). The high base scores in sequencing reads further supported the high quality the of PHDs-seq library (Fig. S1D). We next conducted hierarchical clustering and correlation analysis based on the transcriptional profiles of the probed gene set. Clustering analysis showed that PHDs-seq could distinguish KF from positive samples at both the D5 and D8 time points, which was further confirmed by correlation analysis (Fig. 2B and C). Further analysis showed a high correlation (R^2^ = 0.9542) in both the low (*PRDM16, UCP1, EBF2)* and high abundance genes (*ADIPOQ, COL1A1, ACTB, GAPDH, THY1)* for which two probe pairs were used, thus demonstrating the high sensitivity of the PHDs-seq platform (Fig. 2D). Additionally, analysis of consistency confirmed the similarity of the mRNA profiles between parallel replicates of positive samples, with a fitted equation of y = 1.0579x-0.18686 (R^2^ = 0.97926). These results indicated the high reproducibility of PHDs-seq (Fig. 2E). Following this validation process, we then compared the relative levels of several representative signature markers detected by PHDs-seq with that determined by qPCR assays of D5 and D8 samples (Fig. S1 E-H). The results illustrated high similarity in the detected fold change of most markers between the two methods (R^2^ = 0.8781 at D5 and R^2^ = 0.9272 at D8), further showing the high accuracy of PHDs-seq (Fig. 2 F and G). These results, with validation, indicated that the PHDs-seq platform was a highly sensitive, reproducible and accurate for evaluating cell status or fate through the detection of several groups of signature transcripts.

### Signature index (SI) for assessing candidate drugs in PHDs-seq data

**Fig. 3:**
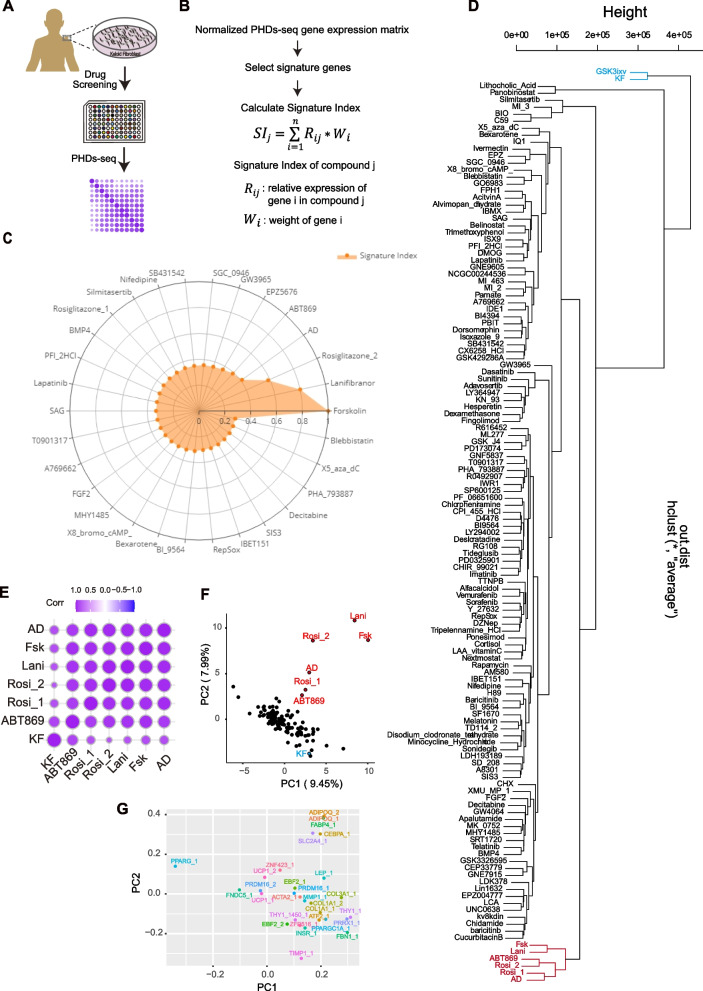
PHDs-seq screen to discover candidate drugs that promote adipocyte reprogramming. **A** Schematic diagram of steps in the PHDs-seq screening process. **B** Schematic for the calculation of signature index (SI). **C** Radar map of the adipocyte SI for top30 samples. Sample names are compound names. **D** Hierarchical clustering of PHDs-seq data from 128 compounds and control treatments. Blue indicates negative control KF samples or ineffective compounds; red indicates AD positive controls or candidates; _1 and _2 represent biological replicates. **E** Pearson correlation analysis of candidates, AD, and KF data from PHDs-seq. **F** PCA analysis of PHDs-seq data from 128 compounds and control treatments. **G** PCA analysis of the major contributing genes

Having assessed the performance of PHDs-seq, we next investigated the use of PHDs-seq in screening for compounds that efficiently induce adipocyte reprogramming. Previous reports have shown that triiodothyronine and rosiglitazone in AD medium were essential components for brown adipocyte differentiation (Long et al. 2014; Ohno et al. 2012; Park et al. 2013; Rosen and Spiegelman 2014; Tu et al. 2019). To evaluate the roles of each compound in drug screening, we used MDI medium (DMEM/high glucose, 1% ITS, 0.5 mM isobutylmethylxanthine, 1 μM dexamethasone) as a basal medium to screen 128 compounds (2 μM) known to be involved in regulating signalling pathways such as Tgfβ/BMP/SMAD inhibitors, JAK-STAT inhibitors, MEK inhibitors, GSK-3α/β inhibitors, AMPK activator, cAMP agonists, epigenetics related compounds, some kinase inhibitors, etc. (Fig. 3A and Table S2). After 4 days of culture, the medium was changed to DMEM/high glucose with 1% ITS, and cells were cultured for another 4 days. The negative controls were KF cells grown in KF medium for 8 days, while positive controls were KF cells grown in AD medium for 4 days, then AM medium (DMEM/high glucose, 1% ITS, 0.1 μM cortisol, 0.2 nM triiodothyronine) for another 4 days. Cells were harvested for PHDs-seq library preparation at day 8.

Algorithm development was valuable in analysing gene expression data for stem cell research, especially for cell reprogramming. However, for targeted sequencing methods, no algorithm was reported to evaluate the cell fates/states of the samples after drug treatments. To facilitate the analysis of low abundance genes that are crucial for cell fate transition in signature gene sets, we developed an algorithm to adjust for the contributions of transcription factors by considering both the relative expression and weight of each gene. Specifically, the algorithm calculated the relative expression (*R*_*ij*_) for each gene *j* and then calculated the sum of product of *R*_*ij*_, with the weight of each gene (*W*_*i*_) to enrich the contribution of differently expressed genes in the assessment of cell fates/states_*,*_ hereafter called the “signature index (SI)” of each sample (Fig. 3B, see Methods for details). At last, SI was adjusted to 0–1 through dividing by the maximum of SI value among all sample. With SIs of positive and negative controls (AD and KF samples) (Tables S4, and S5), we obtained a Z’ value as 0.32233, indicating the acceptable robustness and reproducibility of this assay quantified by PHDs-seq and SI algorithm (Birmingham et al. 2009; Zhang et al. 1999).

Next, SI value was then used to evaluate the overall effect of each compound in inducing reprogramming. We calculated the SI for each compound and noticed that the three highest ranking compounds, including Forskolin (Fsk, an activator of adenylate cyclase that increases cAMP levels) and two agonists of peroxisome proliferator-activated receptor (PPAR), Rosiglitazone (Rosi) and Lanifibranor (Lani), had higher SI than positive control AD (Fig. 3C). In addition, we found that the compound ABT869 also had a similar SI to that of the AD controls. To further validate the reliability of SI, we performed hierarchical clustering and principal component analysis (PCA). The results showed that these compounds with high SI indeed clustered together with AD sample (Fig. 3 D-F).

Subsequent analysis of reads from all probed genes revealed that the clustering patterns in PC1 and PC2 could be largely explained by the expression of adipocyte signature genes, including *ADIPOQ, FABP4, CEBPA,* and *SLC2A4*, rather than by fibrosis signature expression (Fig. 3 G). These results showed that the SI algorithm could successfully identify candidate drugs using PHDs-seq data and that PHDs-seq screening could validate the effects of known compounds as well as discover new compounds in high-throughput drug screens.

### Functional confirmation of drug candidates identified by PHDs-seq

**Fig. 4:**
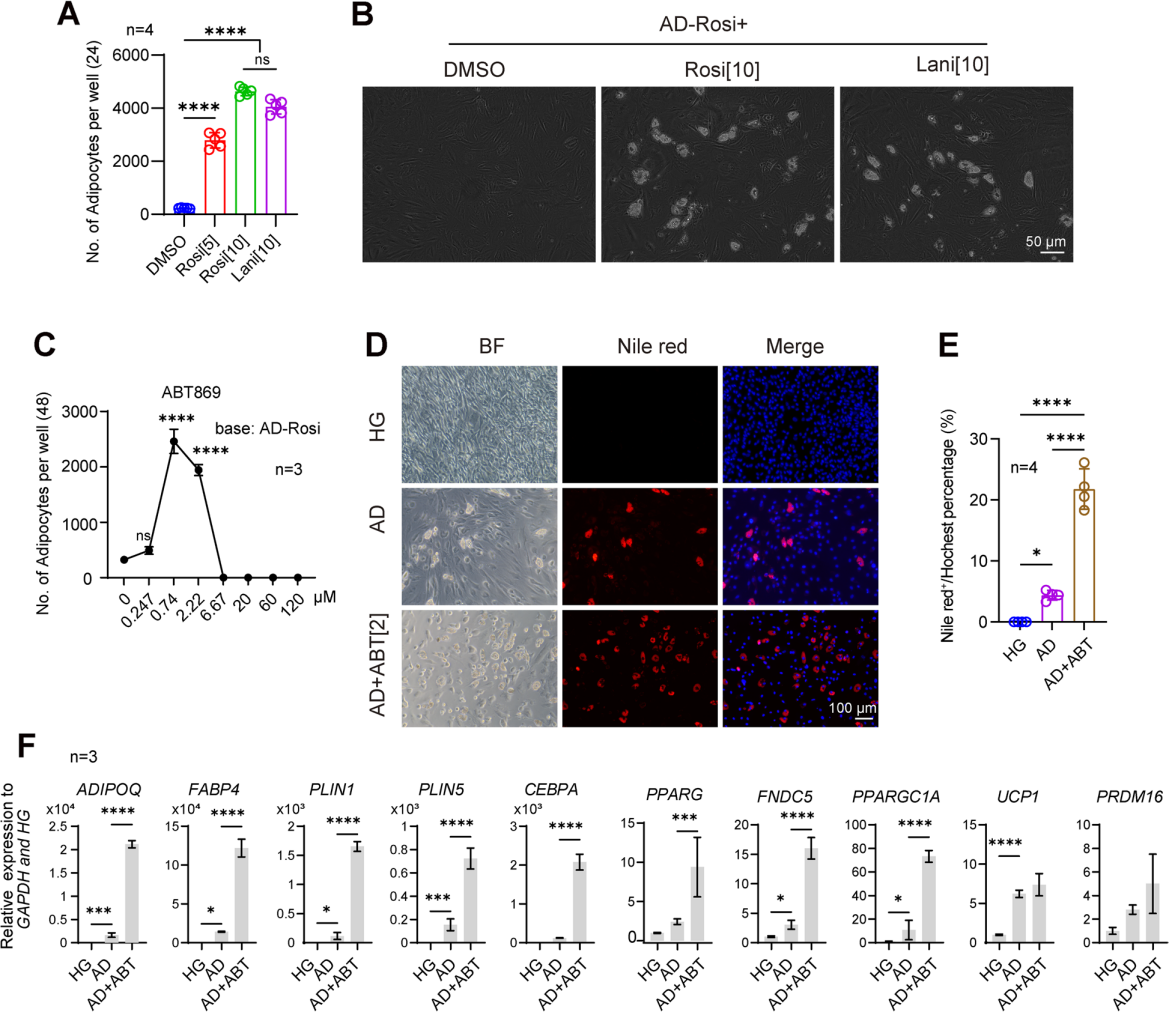
Lanifibranor and ABT869 promote adipocyte reprogramming. **A** Quantification of adipocyte induction in presence of Rosi or Lani in AD medium minus Rosi; **** *p* < 0.0001, *** *p* < 0.001, ns, *p* > 0.05 between the indicated groups, biologocal replicates: 2, numbers in square brackets indicate concentration in μM. **B** Reprogrammed adipocyte morphology of cells in (A). **C** Adipocyte reprogramming dose response curve for ABT869 in AD medium minus Rosi, biologocal replicates: 3, **** *p* < 0.0001, ns, *p* > 0.05 between indicated groups. **D** Nile red staining for lipid in adipocytes (red) or Hoechst (blue). ABT: ABT869; concentration: 2 μM; scale bar = 100 μm; HG, DMEM/high glucose medium. **E** Statistical analysis of adipocyte percentages per field in (D), fields: 4, **** *p* < 0.0001, * *p* < 0.05 between indicated groups. **F** qPCR quantification of the indicated adipocyte marker expression levels. biologocal replicates: 3, **** *p* < 0.0001, * *p* < 0.05 between indicated groups. Statistical methods: One-way Analysis of Variance (ANOVA) in (A)(C)(E)(F)

Among the four candidates, Fsk had a similar function to that of isobutylmethylxanthine in increasing cAMP levels. Notably, isobutylmethylxanthine and Rosi were both components in AD medium that reportedly facilitate adipocyte differentiation (Plikus et al. 2017; Tu et al. 2019). In addition, Lani and Rosi share a common target PPARG. Based on this understanding, we therefore confirmed that both Lani and Rosi could promote adipocyte reprogramming from KF (Fig. 4 A and B). Next, we over-expressed *PPARG* in KF cells cultured in KF medium and discovered that *PPARG* over-expression could substantially improve adipocyte induction rates via upregulation of adipocyte-related transcription factors and maturation markers (Fig. S2).

To verify that compound ABT869, discovered through PHDs-seq, indeed functioned in adipocyte reprogramming, we conducted dose curve assays and found that the optimum concentration range was 0.74–2.22 μM (Fig. 4C). Therefore 2 μM ABT869 was used in subsequent experiments investigating its effects on adipocyte induction in AD medium. Nile red staining for lipids (Fig. 4D, quantified in E) and qPCR of adipocyte markers (Fig. 4F) showed that adipocyte reprogramming efficiency was significantly higher in the presence of ABT869 than that in controls due to enhanced expression of developmental transcription factors and functional markers, including some brown adipocyte markers (*PPARG, PPATGC1A, FDNC5*).

The cumulative data experimentally confirmed that the candidates Rosi and Lani could indeed promote adipocyte reprogramming. This effect was verified by overexpression their target, PPARG, which also increased the efficiency of adipocyte induction, supporting that the PPARG pathway was responsible for mediating their activity. Finally, ABT869 was also verified to promote adipocyte reprogramming. These results indicated that candidates identified in PHDs-seq data using the SI algorithm were effective for adipocyte reprogramming, thus providing a proof-of-concept demonstration of the accuracy and reliability of the PHDs-seq platform in drug discovery.

## Discussion

In this work, we developed the PHDs-seq platform for drug screening based on targeted sequencing of signature genes. PHDs-seq provides several advantages over RASL-seq (Larman et al. 2014), MLPA-seq (Kondrashova et al. 2015), TempO-Seq (Yeakley et al. 2017), and TAC-seq (Teder et al. 2018), although these platforms all share similarities in the underlying principle of probe hybridization and ligation-based amplification in conjunction with targeted transcriptional profiling. First, PHDs-seq was performed using whole cells cultured in 96-well plates rather than mRNA, RNA/DNA hybrids, or DNA, as in the above sequencing methods. Although RASL-Seq can also be performed with whole cell cultures, it requires oligo(dT) magnetic beads to enrich mRNA templates from cell lysate, which is not easily available for most labs, and this step can lead to variabilities due to potential RNA degradation. In comparison, PHDs-seq is much easier by using reverse transcription derived cDNAs as hybridization templates, and more stable than RASL-seq in principle. We developed a mild lysis buffer, compatible with the reverse transcription reaction, to release mRNA from cells, thereby considerably reducing time, costs, and operational steps, and enabling large-scale drug screening. Second, probe hybridization and ligation were simplified into a single step in PHDs-seq by optimizing a buffer originally described for use in TAC-seq. Third, the PHDs-seq library structure was designed to allow paired-end barcoding that includes a well barcode to distinguish samples in the same plate and a plate barcode to identify different plates in large-scale screens. These optimizations thus ensure that libraries of 2–4 96-well plates could be prepared within one day at a current cost per sample of < $1.5 USD (or 10 RMB). It’s reported that RASL-seq can be carried out in 384 or 1563-well plate formats (Larman et al. 2014). In principle, if 384/1563-well plate magnet and 384 or 1563-well PCR instrument are available, PHDs-seq can also be carried out in 384 or 1536-well plate formats, similar to RASL-Seq.

In addition, it’s reported that DRUG-seq was developed to mine chemical compounds via high throughput detecting genome-wide transcriptional profiling (Ye et al. 2018). We noticed that DRUG-seq approach was more inclined to easily detect highly-expressed genes, while it was not sensitive to detect genes with low abundance. By contrast, PHDs-seq had the superiority in detecting low-expression genes. For example, *PPARG* and *PPARGC1A* were detected by PHDs-seq (Table S3), while DRUG-seq approach did not detect these two genes (data not shown) even when more than 10,000 genes were detected in that assay, suggesting that PHDs-seq had advantages over DRUG-seq in sensitivity.

Although, ideally, PHDs-seq can be used as a high-throughput transcriptomic drug screening system, only < 200 chemicals in one batch were screened in this proof-of-concept demonstration, and thus the current study does not fully illustrate the PHDs-seq capacity for scalability, time reduction, or cost-effectiveness. However, in our own future work as well as other groups, it is likely that PHDs-seq will be commonly used for larger scale drug screens.

At the data mining level, the SI algorithm is suitable for evaluating the contribution of a group of signature genes (as in PHDs-seq) rather than a genome-wide expression profile because it emphasizes the importance of all signature genes including low and high abundance ones. By contrast, in stem cell research, the cellNet algorithm is well-suited for calculating cell fate scores because it utilizes cell-type gene regulatory networks which require whole transcriptome expression profiles (Cahan et al. 2014). It is to be investigated whether cell-type gene regulatory networks can be integrated with PHDs-seq data analysis in the future.

Using the SI algorithm to sift PHDs-seq data, we found several compounds targeting the previously known pathway in inducing adipocytes, including Fsk, Rosi, and Lani. More importantly, we identified a new compound, ABT869, that promotes adipocyte reprogramming by activating *PPARG, PPARGC1A, FDNC5*. ABT869 is a potent ATP-competitive inhibitor of vascular endothelial growth factor (VEGF), platelet derived growth factor (PDGF) family receptors (e.g., KDR), and colony-stimulating factor-1 receptor (CSF-1R) (Albert et al. 2006; Guo et al. 2006). ABT869 exhibits anti-proliferative effects in multiple cancers with FMS-like tyrosine kinase 3–internal tandem duplication (FLT3-ITD) (e.g., acute myeloid leukemia) by inhibiting phosphorylation of FLT3, STAT5, and ERK, consequently inducing apoptosis (Shankar et al. 2007). Thus, ABT869 is currently under clinical development as an intervention for some types of carcinomas, such as non-small-cell lung cancer, hepatocellular carcinoma, acute myeloid leukemia, and metastatic colorectal cancer (Cainap et al. 2015; O'Neil et al. 2014; Posadas et al. 2011; Ramalingam et al. 2015; Tannir et al. 2011). In the field of cell reprogramming, ABT869 is used to synergistically induce an intermediate plastic state before the XEN-like colony stage during human pluripotent stem cell formation (Guan et al. 2022). In this study, we found that ABT869 could also promote adipocyte reprogramming from fibroblasts. Earlier studies have shown that PDGFRα/PDGFRβ are both negative regulators of adipogenesis (Sun et al. 2020). Consistent with this function, blocking PDGF-induced PI3K activity with imatinib promotes adipogenesis (Fitter et al. 2012), suggesting that the PDGFR pathway probably contributes to adipocyte reprogramming. However, whether the mechanisms of CiPSC and adipocyte reprogramming are similar to that in cancers (i.e., via inhibition of VEGF/PDGFR/CSF-1F/FLT3) requires further investigation.

ABT869 was also reported to promote adipocyte browning and suppress the differentiation of pre-adipocyte 3T3-L1 (an immortalized mouse cell line), through inhibiting STAT3 signalling pathway (Yi et al. 2019). In comparison with this study, we carried out experiments mainly on primary human fibroblasts. Interestingly, ABT869 indeed upregulated some markers of brown adipocyte, such as PPARG, FNDC5, and PPARGC1A, in our study (Fig. 4F).

## Conclusions

We established the PHDs-seq high-throughput transcriptional signature drug screening platform and the concomitant SI algorithm for data analysis. Using PHDs-seq with the SI algorithm, we found several known compounds that can induce adipocyte formation, including Forskolin, Rosiglitazone and Lanifibranor. More importantly, our proof-of-concept screen identified a new compound, ABT869, that promotes adipocyte reprogramming, indicating the potential for further development of ABT869 as a small molecule drug lead for use in cell reprogramming, as well as an effective approach to identify chemical regulators in the field of stem cell and reprogramming.

## Methods

### Isolation and culture of KF

Adult human KF tissues were obtained with informed written consent and approval by the Institute of Ethics Committee Review Board in Chinese Academy of Medical Sciences and Peking Union Medical College (ethics approval number: 2022-KY-043). Adult human KF tissues were soaked in 70% ethanol for 1 min, washed buffer (PBS containing 2% penicillin–streptomycin) then minced by scissors. The minced tissues were then transferred to a 10 ml wash buffer, mixed well, and centrifuged at 1500 rpm for 3 min. The collected tissues were dissociated with 10 ml digestive solution (1 mg/ml Dispase II, 1 mg/ml collagenase II, 0.167 U/μl Dnase I, 2% penicillin–streptomycin and 10 μM Y-27632) in a 10 cm dish at 37 °C for 3 h. 10 ml of 10% FBS-DMEM containing 10 μM Y-27632 of medium was added. The cell mixture was transferred to a sterile conical flask and incubated in a magnetic stirrer for 2 h. Next, the cell suspension was filtrated over a 70 μM filter and centrifuged at 1500 rpm for 8 min. The collected cells were resuspended in 1 ml of KF medium (10% FBS DMEM, 2% penicillin–streptomycin, 1% ITS, and 20 ng/ml bFGF). 3 ml of red blood cell lysis buffer was added. Cells were incubated on ice for 15 min and then centrifuged at 1500 rpm for 8 min. After removing the supernatant, cells were plated in a 10 cm dish with KF medium and incubated under 5% CO_2_ at 37 °C. The KF medium was changed every 3 days. KFs usually became confluent within 7 days. For cell passage and collection, 0.25% trypsin–EDTA was used to dissociate KFs. Cells were centrifuged and collected then frozen in liquid nitrogen. KFs used for adipocyte reprogramming were within 10 passages.

### Generation of adipocytes from KF

For adipocyte induction, KFs were seeded at a density of 8 × 10^4^ cells per well of a 12-well plate containing KF medium. The next day, KFs were incubated in AD medium (DMEM/high glucose, 1% ITS, 0.5 mM isobutylmethylxanthine, 0.1 μM cortisol, 1 μM dexamethasone, 0.2 nM triiodothyronine, 1 μM rosiglitazone) or AD medium plus ABT869 for adipocyte fate transition. After 4 days, the AD medium was changed to an AM medium (DMEM/high glucose, 1% ITS, 0.1 μM cortisol, 0.2 nM triiodothyronine) for adipocyte cell fate maintenance for another 8 days. The AM medium was changed every 4 days. Cells were harvested on day 12 for further identification experiments.

### Small molecule screening

For small molecule screening, 128 compounds were collected based on functions in the signaling pathway and cell fate transition. KFs were seeded at a density of 0.5 × 10^4^ cells per well of a 96-well plate containing KF medium. The next day, the medium was changed to MDI medium (DMEM/high glucose, 1% ITS, 0.5 mM isobutylmethylxanthine, 1 μM dexamethasone) plus 2 μM compounds. After 4 days, DMEM/high glucose with 1% ITS was added to the culture for another 4 days. The negative control was KF in KF medium for 8 days. The positive control was KF in AD medium for 4 days and then AM medium for another 4 days. Cells were harvested on day 8 for PHDs-seq library preparation.

### Probes design for PHDs-seq

We designed probes (including left and right probes) as previously described with a slight modification (Wang et al. 2017). The rules include: 1) 27 nts length, span exons; 2) GC content is between 40%-60%; 3) No or limited homology with other human sequences; 4) No SNP in the ligation site.

### PHDs-seq library preparation

Samples with compound treatments in a 96 well-plate were washed with pre-warmed PBS. 60 μl of lysis buffer containing 50 mM Tris–HCl pH8.0, 75 mM KCl, 6% Ficoll PM-400, 0.15% TritonX-100, and 0.5U/μl RNaseOUT (RNaseOut should be added before use) were added to each well. The 96-well plate was then incubated on a shaker at 900 rpm for 15 min. 4.286 μl of cell lysate were transferred to a new 96 well PCR plate. 0.714 μl reverse transcription mix containing 40 mM MgCl_2_, 4 μM template switching oligos (TSO), 0.1 mM dNTP, 8U/μl Maxima reverse transcriptase, 1 × ERCC, and RNaseOUT (RNaseOut should be added before use) were added to each well, mixed and slightly centrifuged for several seconds to collect all reactants on the bottom. The 96-well PCR plate was incubated at 42 °C for 2 h with a hot lid at 105 °C. 6 μl of hybridization-ligation mix (0.005 μM probe mix, 1 U/μl Taq DNA ligase, 1 × Taq DNA ligation buffer) was added to the RT products, mixed gently, and incubated at 60 °C for 60 min with a hot lid of 105 °C. After hybridization and ligation, the products were enriched with 15 ul of enrichment mixture (2 μl Dynabeads MyOne Carboxylic Acid beads plus 13 μl capture buffer containing 30% PEG-600, 2 M, NaCl, 5 mM Tris–HCl pH7.5, 10 mM EDTA, and 0.02% Tween-20). Next, 20 μl of PCR mixture (0.25 μM PHD-seq well barcode, 0.5 mM PHD-seq plate barcode, 1 × Vazyme LAmp Master Mix) were added to the enriched hybridization-ligation products, mixed, and incubated at 94 °C for 5 min, followed by two cycles of 95 °C for 20 s, 57 °C for 30 s and 72 °C for 20 s. In addition, 10 cycles of 94 °C for 20 s, 65 °C for 30 s, and 72 °C for 20 s with a final extension at 72 °C for 60 s. The PCR products were pooled together into several 1.5 ml tubes which were placed on a magnet to remove the beads. The supernatant was purified with DNA Clean & Concentrator-100 column (Zymo Research, D4029) and eluted with 150 µl of ddH_2_O. The library was size-selected using AMPure XP beads. 150 µl of beads were added to 150 µl of the purified PCR product, incubated for 5 min, and captured by a magnet for 3 min after which the supernatant was discarded. The beads were eluted in 25 µl of ddH_2_O and incubated for 1 min at room temperature. After 1 min of incubation on the magnet, the supernatant was transferred to a clean 1.5 ml tube. The 208 bp library was visualized using agarose gel electrophoresis and quantified using a Qubit™ 4 NGS Starter Kit with WiFi (Q33240).

### PHDs-seq data analysis

PHDs-seq libraries were sequenced by Illumina HiSeq X-Ten instrument. Data were analysed as previously described (Teder et al. 2018). First, sequencing reads were mapped to the reference sequences (left probes plus right probes) to produce total counts of each gene. The CPM (Counts of exon model per million mapped reads) matrix was then transferred from the count matrix and used in cluster analysis, Pearson correlation analysis, and PCA analysis.

### Signature index

To calculate the signature index, we first obtained the gene expression of the signature genes. We defined the gene expression in KF as the starting point of reprogramming and that in AD as the endpoints. Next, we calculated the relative expression of each gene i in each compound j as *R*_*ij*_ = *(G*_*ij*_* – Gi*_*KF*_*) / (Gi*_*AD*_*– Gi*_*KF*_*)*, where G denotes normalized gene expression (CPM). Considering that each gene contributes differently to the cell fate, we assigned a weight of each gene *W*_*i*_ to increase the contribution of differently expressed genes to SI value: *W*_*i*_ = abs [log_2_*(Gi*_*AD*_*/ Gi*_*KF*_*)*]. Then for compound j containing n genes, we calculated the signature index (*SI*_*j*_) as the weighted sum of relative gene expressions across genes:

$${{\varvec{S}}{\varvec{I}}}_{j}={\sum }_{i=1}^{n}\left({{\varvec{R}}}_{ij}*{{\varvec{W}}}_{i}\right)$$

Here, ***SI*** could sum the contribution of gene expression with similar pattern to the positive control AD and simultaneously minus that with opposite pattern to AD. At last, SI was adjusted to 0–1 through dividing by the maximum of SI value among all sample.

### Nile red staining

Cells were washed with PBS and then incubated in 1 μg/ml Nile Red and Hoechst for 10 min at 37 °C. After staining, the cells were washed again and snapped at 488 nm with a fluorescence microscope (ZEISS, AXIO).

### Quantitative PCR

Total RNA from cells was extracted with EasyPure RNA Kit (Transgene, ER101-01) according to the manufacturer’s instructions. 1 μg of total RNA was reverse-transcribed to cDNA using transScript One-Step gDNA Removal and cDNA Synthesis SuperMix (Transgene, AT311-02) at 42 °C for 30 min, and at 85 °C for 5 s to deactivate enzymes. Quantitative PCR (qPCR) reactions were performed using ChamQ SYBR qPCR Master Mix (Vazyme, q321-03) in a KUBO thermocycler system (KUBO, Q225). The primer sequences for RT-qPCR are listed in Table S1.

### Statistical analysis

Unpaired Student's t test was utilized to compare the significant difference between two groups. One-way Analysis of Variance (ANOVA) was used to compare the significant differences among more than three groups. All analyses were performed on GraphPad Prism 9.0.0.121 (GraphPad Software, San Diego, CA).

## Supplementary Information

**Additional file 1:** **Fig.S1.** Establishment and evaluation of PHDs-seq.

**Additional file 2:** **Fig.S2.** Overexpression of PPARG promoted adipocyte reprogramming.

**Additional file 3:** **Table S1.** Oligos or primers used in this study.

**Additional file 4:** **Table S2.** Small molecules information and SI Scores.

**Additional file 5:** **Table S3.** PHDs-seq_CPM.

**Additional file 6:** **Table S4.** CPM of AD and KF.

**Additional file 7:** **Table S5.** Z' value.

## Data Availability

Figure S1-2 and Table S1-5 are provided with this paper. Supplementary sequencing data are available in Gene Expression Omnibus database under accession codes GSE227387, GSE227388, GSE227391, and GSE227392. Any additional information required to reanalyze the data reported in this paper is available from the lead contact upon request.

## Abbreviations

PHDs-seq: Probe Hybridization based Drug screening by sequencing
KF: Keloid fibroblasts
AD: Adipocyte Differentiation medium
UMI: Unique Molecular Identifier
SI: Signature Index
Fsk: Forskolin
Rosi: Rosiglitazone
Lani: Lanifibranor
ABT: ABT869
PCA: Principal Component Analysis
TSO: Template Switching Oligos
R_ij_: Relative expression of each gene i in each compound j
*W*_*i*_: Weight of each gene
PPAR: Peroxisome Proliferator-Activated Receptor
MOA: Mechanisms of Action
