# Supplementary figures and images for: PHDs-seq: a large-scale phenotypic screening method for drug discovery through parallel multi-readout quantification
Source: Cell Regen. 2023 Jun 2;12:22. doi: 10.1186/s13619-023-00164-9 (PMC10235360; doi:10.1186/s13619-023-00164-9)

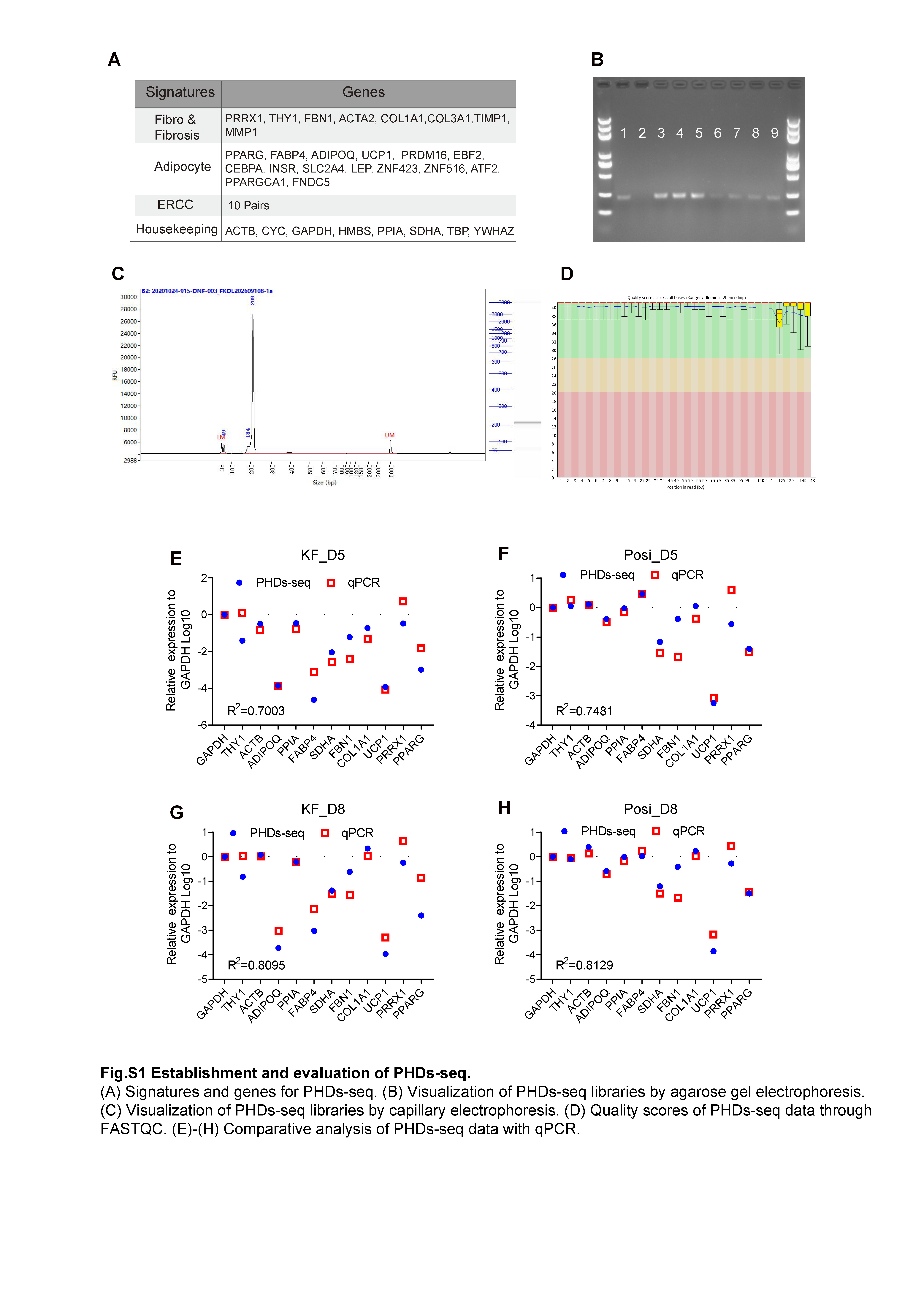

Supplement: Supplementary file 1 — Additional file 1: Fig.S1. Establishment and evaluation of PHDs-seq. [file 13619_2023_164_MOESM1_ESM.tiff]

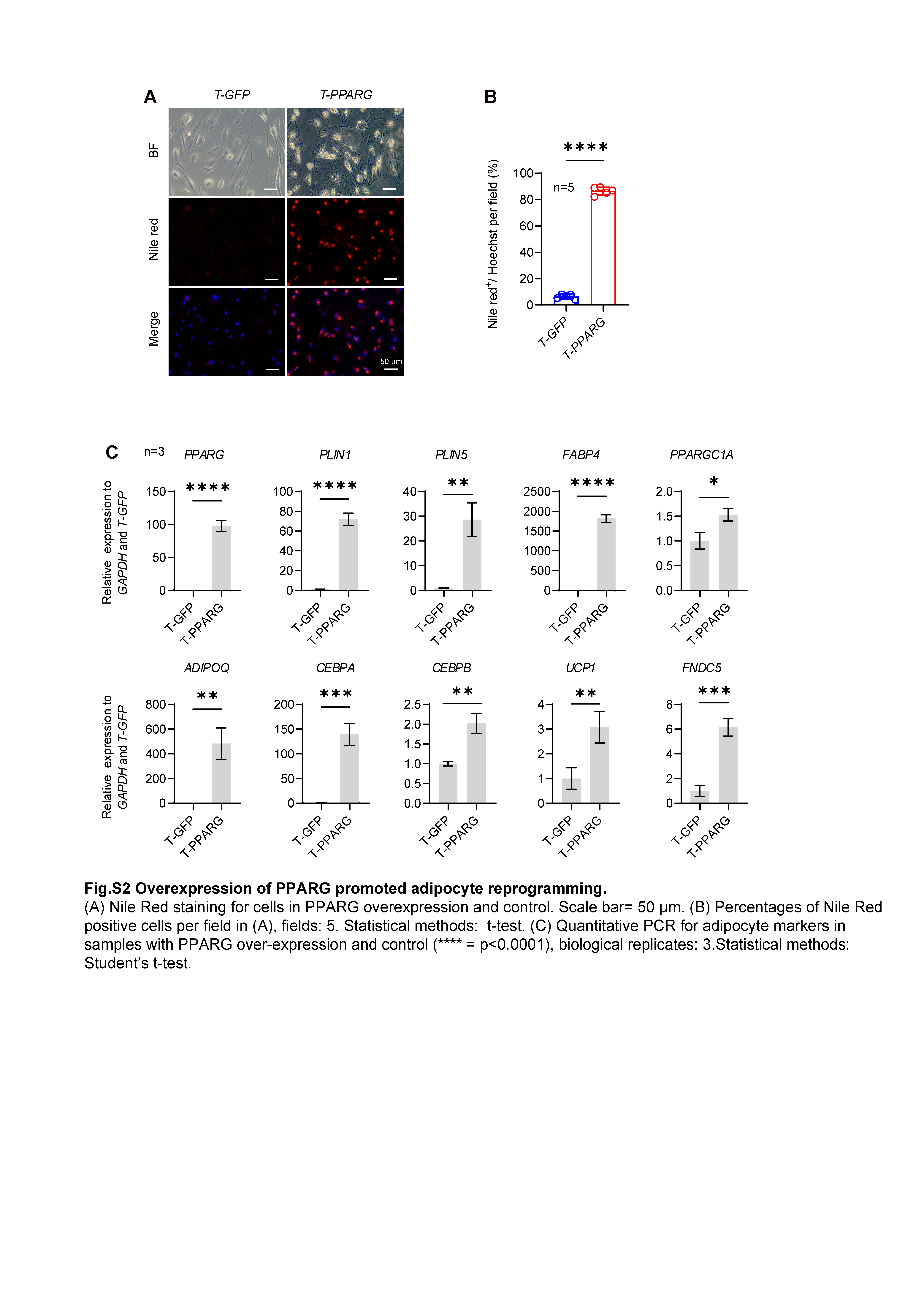

Supplement: Supplementary file 2 — Additional file 2: Fig.S2. Overexpression of PPARG promoted adipocyte reprogramming. [file 13619_2023_164_MOESM2_ESM.tiff]
